# Cholera

**Published:** 1864-10

**Authors:** 


### *Cholera.*

At a meeting of the Harveian Society, Dr. Toulmin, of Brighton, read a paper *on the pathology of cholera, with an exposition of the causes which necessarily render medicines inoperative in that disease* He commenced by remarking that, in medicine as in other sciences, dogmas were sometimes received as truths without having been proved to be true, and that this sadly impeded the advancement of knowledge. That in his present inquiry into the phenomena of cholera, he found one of these time-honored beliefs obstruct his progress, and which it became necessary to refute before he could expect his theory of the disease would be honored with the approbation of the Society. The dogma he referred to was, that in all cases death must necessarily ensue on the cessation of respiration or the circulation of the blood. To this he objected to this extent—viz: that where black blood was not being carried to the lungs or brain, death did not necessarily ensue on the cessation of those functions, but that a passive state of existence might continue for many hours, or even days, after these functions had completely ceased. As a proof of this, he instanced the act of fainting (which sometimes was of long continuance), drowning, the hybernation of animals, and more particularly the state of trance, of which he read the curious and interesting case of the Hon. Colonel Townshend, as given by Dr. Cheyne. He admitted that life consisted in a never-ending series of phenomena, not one of which could be arrested singly without the consequent death of the individual; but he proposed a case where, in full health, all the functions should be simultaneously suspended—where, although respiration had ceased, no carbonized blood was suffused on the lungs or brain, because the veins had ceased to carry any—where, although the secretion of urine was stopped, still no uremic poisoning ensued, because there was no metamorphosis of tissue going on to produce it; and then asked, what was there in such a case to produce immediate death? Life might be suspended, but not lost; and he contended that in a similar state to this persons continued to exist for a certain time in the collapse of cholera, and were thus frequently buried alive.

He then described cholera as a certain poison, *sui generis*, which was received into the blood, and which attached itself particularly to the serous portion of the fluid; but observed, that however virulent the nature of the poison might be, still all the subsequent phenomena, terminating even with the death of the individual, arose from a series of consecutive causes of the most simple and natural kind, originating in nature's efforts to expel this poison from the system through the medium of the alimentary canal—the rice-water evacuations being, confessedly, nothing more than serum derived from the blood From these facts he drew the following important deduction—viz: that any attempt to arrest the diarrhœa of cholera was not only futile, but mischievous.

He then observed that all the subsequent symptoms of the disease might be traced to this one conservative act of nature: as, first, the rapid cooling of the body, which arose from the absolute cessation of all combustion of matter; that this again was caused by all metamorphosis of tissue being arrested, which in like manner depended on the sudden coagulation of the blood, as the necessary consequence of the sudden and total loss of all its serum the very celerity of which cut off all further supply. The absolute cessation of the circulation of the blood, he remarked, was due to this one cause, conjoined to another, either of which was equal to the occasion—viz: the shock given to the heart's action at the moment the poison was first received into the system.

The author then remarked, that in such a state as this the patient ceased to be governed by the laws of animal life and that the attempt to keep him warm, after his own power of generating heat was lost, by wrapping him in blankets, was as unphilosophical as it would be with a marble statue.

As a demonstrative proof of the absolute stagnation of the blood, and that all change of matter depending thereon had ceased, he instanced the state of respiration, the air inspired returning unaltered; the rapid loss of heat, as well as the power of generating it; the arrest of the secretion of urine, as well as of bile, and indeed of all functional activity. As a proof of the latter, he noticed that mercury never produced ptyalism, nor opium sleep; that calomel, when given, was found adhering to the lining membrane of the stomach unabsorbed; and he believed that in the state of collapse any poison might be given (not acting mechanically) with impunity, until re-action was again established, when its specific effect would for the first time become apparent, to the serious detriment of the patient.

The extreme thirst always attending on cholera he explained as the inevitable consequence of the great loss of fluid in the system; and cramp, as urea deposited among the fibrillæ of the muscles before combustion had entirely ceased, remaining unabsorbed.

In considering the treatment of the disease, he divided the period of its existence into two stages: the first (and where medical treatment was all-important) extending to the commencement of collapse; the second as being the state of collapse itself, to which alone all his observations had reference, and in which the exhibition of medicine would be as useless as it would be in giving it to an automaton. The author then asked what, pathologically speaking, the system most loudly called for? The answer to which, not only from science, but also from the patient himself, was—*water*, to dilute the crassamentum, and thus re-establish the circulation of the blood, which, when once effected, life was saved. But how, he enquired, was this to be effected when all power of absorption was lost? By the endosmose of tissue, and by this alone. By this function, he observed—which plays so important a part in the animal economy, and, as is well known, is equally active in dead as in living membrane—the fluid swallowed is, in the most direct and simple manner, at once imbibed in its course through the alimentary canal by all the parts requiring it—by the coats of the blood-vessels, the areolar tissue, the abdominal cavities, and the viscera in general; and thus the whole system is supplied with its due degree of moisture, and this without any assistant action on its part. The author pointed out four ways by which water might be imbibed: by the mouth, the skin, the rectum, and by injection into the veins themselves; but only drew the attention of the Society to the first two, which in practice, he observed, he had found all sufficient. By the mouth, the ardent thirst of the patient called for water icy cold, and in fact his whole time should be employed in sucking ice and drinking water; whilst by wrapping him in the hydropathic wet sheet the whole surface of the skin imbibed water in the most subtle and effective manner By these means, he observed, he had never seen a case in which re-action had not been established. At the same time he commented on the potency of the remedy, and urged the importance of seizing the golden moment when re-action was established to remove the patient from the wet sheet, and to place him in a room hotter than his blood, or in a warm bed, and then supplying him with some mild stimulant, in the selection of which he would rather be guided by the wish of the patient than his own fancy.

